# Antiviral Activity of the Human Cathelicidin, LL-37, and Derived Peptides on Seasonal and Pandemic Influenza A Viruses

**DOI:** 10.1371/journal.pone.0124706

**Published:** 2015-04-24

**Authors:** Shweta Tripathi, Guangshun Wang, Mitchell White, Li Qi, Jeffery Taubenberger, Kevan L. Hartshorn

**Affiliations:** 1 Boston University School of Medicine, Department of Medicine, Boston, MA, United States of America; 2 Department of Pathology and Microbiology, University of Nebraska Medical Center, 986495 Nebraska Medical Center, Omaha, NE 68198–6495, United States of America; 3 National Institute of Allergy and Infectious Diseases, Bethesda, United States of America; The Hospital for Sick Children and The University of Toronto, CANADA

## Abstract

Human LL-37, a cationic antimicrobial peptide, was recently shown to have antiviral activity against influenza A virus (IAV) strains in vitro and in vivo. In this study we compared the anti-influenza activity of LL-37 with that of several fragments derived from LL-37. We first tested the peptides against a seasonal H3N2 strain and the mouse adapted H1N1 strain, PR-8. The N-terminal fragment, LL-23, had slight neutralizing activity against these strains. In LL-23V9 serine 9 is substituted by valine creating a continuous hydrophobic surface. LL-23V9 has been shown to have increased anti-bacterial activity compared to LL-23 and we now show slightly increased antiviral activity compared to LL-23 as well. The short central fragments, FK-13 and KR-12, which have anti-bacterial activity did not inhibit IAV. In contrast, a longer 20 amino acid central fragment of LL-37 (GI-20) had neutralizing activity similar to LL-37. None of the peptides inhibited viral hemagglutination or neuraminidase activity. We next tested activity of the peptides against a strain of pandemic H1N1 of 2009 (A/California/04/09/H1N1 or “Cal09”). Unexpectedly, LL-37 had markedly reduced activity against Cal09 using several cell types and assays of antiviral activity. A mutant viral strain containing just the hemagglutinin (HA) of 2009 pandemic H1N1 was inhibited by LL-37, suggested that genes other than the HA are involved in the resistance of pH1N1. In contrast, GI-20 did inhibit Cal09. In conclusion, the central helix of LL-37 incorporated in GI-20 appears to be required for optimal antiviral activity. The finding that GI-20 inhibits Cal09 suggests that it may be possible to engineer derivatives of LL-37 with improved antiviral properties.

## Introduction

Like the defensins, the cathelicidins are a large family of cationic antimicrobial peptides expressed in many species and have broad spectrum antimicrobial activity. Despite this, hCAP18/LL-37 is the only known human cathelicidin [1]. The hCAP18 is 18kD precursor protein with a signal peptide, a cathelin-like domain and antimicrobial domain. LL-37 is a 37- amino acid cationic peptide produced by cleavage of the anti-microbial domain from the hCAP18 protein. Like many other antimicrobial peptides LL-37 is cationic. LL-37 is implicated in host defense against a variety of infections [1–4]. It is produced by neutrophils, macrophages and various epithelial cells as well. LL37 concentration can range from 2–5 μg/ml (0.4–1μM) in bronchoalveolar lavage fluid from healthy individuals and can increase up to 20 μg/ml (2.2μM) during infections. In nasal secretions its concentration can vary from 1.2–80 μg/ml [5, 6]. There is mounting evidence that LL-37 may play a role in host defense against influenza A virus (IAV) through antiviral and immune-modulatory activities. LL-37 improves outcome of IAV infection in mice through inhibition of viral replication and reduction of virus-induced pro-inflammatory cytokine generation [4]. Upregulation of LL-37 expression by stimulation with leukotriene B4 correlated with improved outcome of IAV infection in mice [7]. We have partially characterized the mechanism of anti-IAV activity of LL-37 [8]. LL-37 does not block hemagglutination activity, cause viral aggregation, or reduce viral uptake by epithelial cells, rather it inhibits viral replication at a post-entry step prior to viral RNA or protein synthesis in the cell [8]. Likely sources of LL-37 in the IAV-infected respiratory tract include infiltrating neutrophils [9], macrophages [10] and respiratory epithelial cells [11].

**Fig 1:**
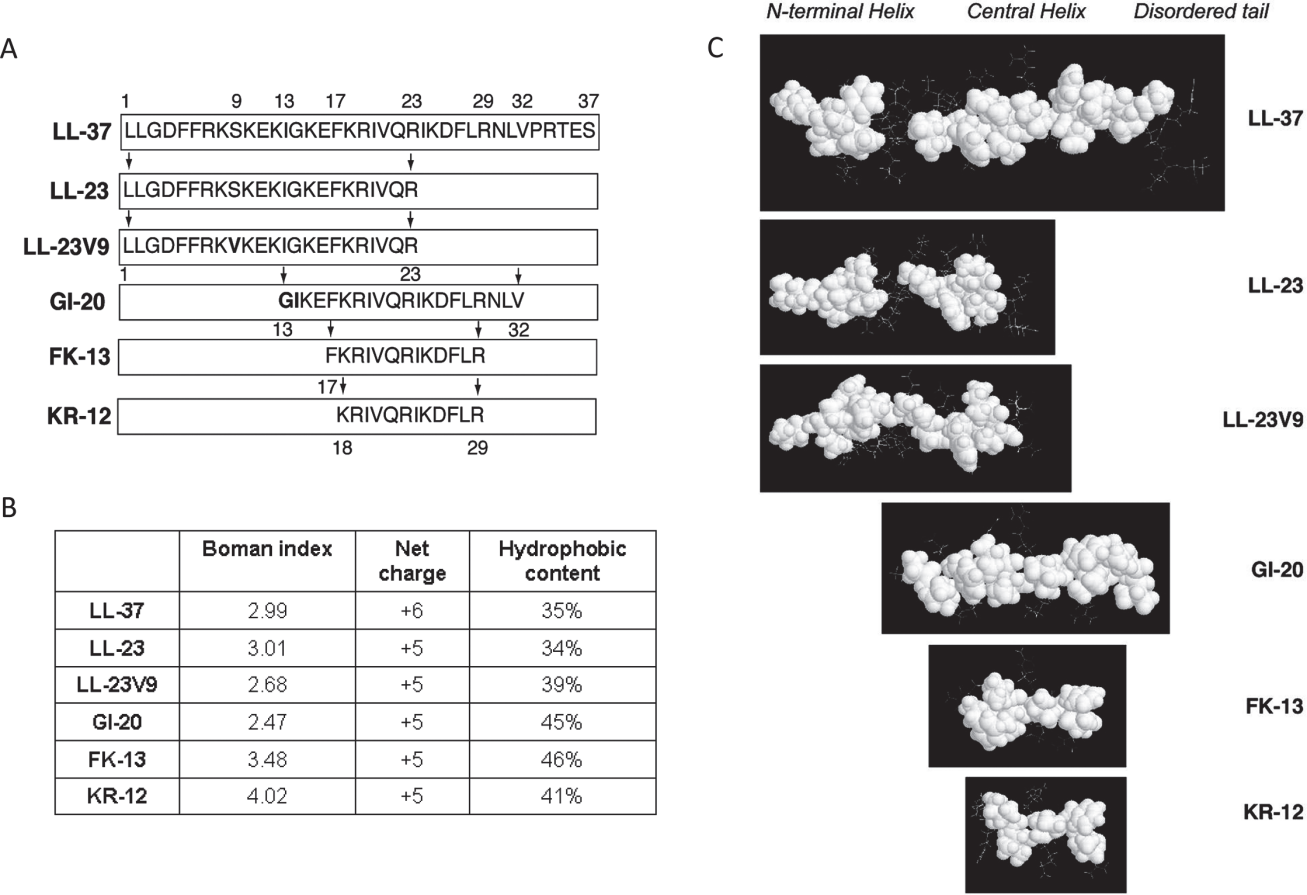
LL-37 and derived peptides employed in this study. Panel A. Shows peptide regions corresponding to the parent LL-37 as indicated with pairs of arrows and residue numbers. Note that GI-20 corresponds to residues 13–32 with the positions of I13 and G14 are swapped (9). In addition, the C-terminus of GI-20, as well as FK-13 and KR-12, is amidated. These LL-37 fragments are named in the same manner as LL-37 by taking the first two amino acids in single-letter code followed by peptide length. Panel B. Biophysical properties of the peptides obtained from or calculated using the Antimicrobial Peptide Database (*http://aps.unmc.edu/AP*). Panel C shows three-dimensional structures of intact LL-37 and its derived fragments. Hydrophobic surfaces are represented with filled space model in white. It is evident that the hydrophobic surfaces of both LL-37 and LL-23 are discontinuous. A mutation of Ser9 to Val9 made LL-23V9 more active against both bacteria [13] and viruses (this study). However, GI-20, corresponding to the central helix of LL-37, had the greater activity against IAV than LL-23V9. These structures are determined by NMR spectroscopy in the presence of membrane-mimetic micelles [28, 29]. The structures of LL-37 and KR-12 are reported in refs. [14], LL-23 and LL-23V9 in ref. [13], GI-20 in ref. [29] and FK-13 in ref [30].

LL-37 is an amphipathic peptide with a predominantly hydrophobic surface and a cationic surface. In addition to LL-37, several active fragments of smaller size are produced in vivo, including LL-23 which contains the 23 N-terminal amino acids of LL-37 [12]. Intensive studies have been undertaken to determine the functional roles of different domains of LL-37 with the goal of developing peptides with increased anti-microbial or immune modulatory activity. Wang et al. has recently shown that LL-23 has limited antibacterial activity and noted that it has a single hydrophilic (serine) interruption in its hydrophobic surface (Fig 1). Replacement of this serine with valine (LL-23V9) significantly improved anti-bacterial activity [13]. The smallest fragment of LL-37 that retains antibacterial activity is KR-12 [14]. This peptide retains the core amphipathic helix structure of LL-37 and carries 5 cationic residues. The slightly larger peptide, FK-13 is the smallest peptide having HIV neutralizing activity [15]. A larger peptide, GI-20 has strong anti-HIV activity comparable to full length LL-37 [15].

For this paper our first goal was comparison of antiviral activity of LL-37 and natural or modified fragments derived from LL-37 against seasonal or mouse adapted IAV strains. Recent studies have shown that some innate inhibitors of seasonal IAV strains fail to inhibit pandemic IAV. These include the collectins, surfactant protein D and mannose binding lectin, and pentraxin [16, 17]. The effects of LL-37 on pandemic IAV have not previously been studied. Hence, our second goal for this paper was to determine the activity of LL-37 and derived fragments (Fig 1) against pandemic IAV.

## Materials and Methods

### Virus Preparations

A/Philippines/2/82/H3N2 (Phil82) strain was kindly provided by Dr. E. Margot Anders (Univ. of Melbourne, Melbourne, Australia). The A/PR/8/34/H1N1 (PR-8) strain was graciously provided by Jon Abramson (Wake Forest University, Winston-Salem, North Carolina). These IAV strains were grown in the chorioallantoic fluid of ten day old chicken eggs and purified on a discontinuous sucrose gradient as previously described [18]. The virus was dialyzed against phosphate buffer saline (PBS) to remove sucrose, aliquoted and stored at -80^°^C until needed. The A/California/04/09/H1N1 pandemic strain (Cal09) and the A/New York/312/01/H1N1 (NY01) seasonal strain were prepared by reverse genetics as described [8, 17]. These preparations contain the intact genome of the original strains. Two additional strains developed by reverse genetics include a strain containing only the hemagglutinin (HA) gene of the pandemic A/Mexico/4108/09/H1N1 combined with the other 7 genes of NY01 (Mex 1:7) and an additional strain containing the HA and neuraminidase (NA) of A/Mexico/09/H1N1 (Mex 2:6). All of the reverse genetic derived strains were grown in MDCK (Madin-Darby canine kidney) cells and the culture supernatants were dialyzed to remove any residual trypsin that might be present.

### LL-37 derived peptides and HNP Preparations

LL-37, FK-13 and KR-12 fragments were purchased from Phoenix Pharmaceuticals, Burlingame, CA. The scrambled LL-37 preparation was purchased from Abgent Inc. LL-23 was purchased from Genemed synthesis (Texas, USA). LL-23V9 and the central fragment of LL-37 (also referred to as GI-20) were developed as described in Dr Wang’s laboratory [13–15, 19]. Human neutrophil peptides defensins 1 and 2 (HNPs 1 and 2) and CRAMP were purchased from Bachem (Torrance, CA).

### Fluorescent focus assay of IAV infectivity

This assay was carried out as previously described [20, 21]. This assay has been used extensively in the literature to study antiviral activity of innate inhibitors [16, 20, 22–24] where it has been shown to predict activity of inhibitors in vivo and on plaque assays [20, 25]. MDCK cell monolayers (American Type Culture Collection, Manassas, VA) were prepared in 96 well plates and grown to confluency. In some assays, normal Human Bronchial/Tracheal Epithelial cells (HBTE) or normal human small airway epithelial cells (SAE) were used. These cells were purchased from the Life Line cell technology (Frederick, MD). All cell lines were propagated in the undifferentiated state in standard tissue culture flasks according to the manufacturer’s instructions. These cell layers were then infected with diluted (in PBS supplemented with Ca^2+^ and Mg^2+^. IAV preparations for 45 min. at 37^°^C (Corning Cellgro, Manassas, VA). Before adding to cell layers, IAV was pre-incubated for 30 min. at 37°C with various concentrations of LL-37 peptides, defensins or control buffer. The multiplicity of infection (MOI) for the fluorescent focus assay and real time PCR (see below) experiments was approximately 0.1. MOI was calculated based on number of cells at confluence. After 45 min, the plate was washed, followed by 24 hrs incubation at 37^°^C in tissue culture media (media used was specific for the cell line used in the experiment and as per manufacturer’s instructions). After 24 hrs, MDCK cells were washed with PBS and fixed with chilled 80% acetone for 10 mins. Presence of IAV infected cells was detected using a primary mouse monoclonal antibody (1::100 dilution) directed against the influenza A viral nucleoprotein (EMD Millipore, MA) as previously described [26]. A rhodamine labeled secondary (1:1000) antibody (EMD Millipore, MA) was used to detect primary antibody. Fluorescent positive cells were counted visually on a fluorescent microscope (Nikon MVI, Avon, MA, US). The number of fluorescent foci per ml (FFC/ml) of inoculum was calculated from this. The raw numbers of positive cells counted per well are given in S1 Data and were comparable for the different viral strains. However, the primary human respiratory epithelial cells were generally less readily infected than MDCK cells. We expressed the data as mean±SEM % of control to make relative comparisons between the peptides. Where differences were statistically significant this was also true when tested using raw numbers of positive cells (data not shown).

### Lactate dehydrogenase (LDH) assay

The LDH assay was performed using LDH cytoxicity detection kit (Clontech, CA, USA) according to the manufacturer’s instructions. In brief, MDCK cells were incubated with Phil82 IAV (with or without LL-37 and related peptides). Cells were also incubated with peptides alone. The incubation time (24 hrs) and assay conditions were exactly same as in fluorescent focus assays. The LDH activity was measured after the 24 hrs incubation of cells with IAV and/or peptides. Controls included uninfected/untreated cells as negative control (NC) and cells treated with lysis solution as positive control (PC). The percent cytotoxicity is obtained from OD values using the formula: [(Sample OD—NC OD) ÷ (PC OD—NC OD)] ×100

### Hemagglutination (HA) inhibition assay

HA inhibition was measured by serially diluting LL-37 peptides in round bottom 96 well plates (Serocluster U-Vinyl plates; Costar, Cambridge, MA) using PBS as a diluent and human type O red cells as described [27]. 40 HA units of virus was used in the assay.

### Plaque assay

The plaque assay was performed as previously described. In brief, IAV (3x10^9^ pfu/ml) was pre-incubated for 30 min. at 37°C with various concentrations of LL-37 peptides, or control buffer, followed by addition of these viral samples to 100% confluent MDCK cells (6 well plates). The IAV samples were prepared in PBS supplemented with Ca^2+^ and Mg^2+^. Cells were infected with IAV samples for 1hr at 37^°^C. After infection cells were washed followed by addition of 2ml agar overlay per well. Composition of agar overlay was as follows: 2X EMEM (Lonza Inc, USA,) with 1% penicillin streptomycin, 4mM L-glutamine (Hyclone), 1% sterile low melting agarose (Fisher Scientific) and 2ug/ml TPCK trypsin. For some experiments IAV was not pre-incubated with peptides before adding to cells. Instead after 1 hr incubation with IAV, cells were washed and then incubated with peptides for another 1hr at 37^°^C followed by washing and addition of agar overlay. After adding agar overlay, cells were incubated for 4 days and then fixed with 4% paraformaldehyde (1hr RT), stained with 0.1% crystal violet and plaques were counted visually.

### Neuraminidase assay

The neuraminidase (NA) assay was performed using a 2'-(4-methylumbelliferyl)-alpha-D-N-acetylneuraminic acid (MUNANA) based influenza neuraminidase kit (Life technologies, USA) as per manufacturer’s instructions. The assay is based on quantitation of fluorogenic end product 4- methylumbelliferone released from non-fluorogenic MUNANA by neuraminidase. Various doses of peptides were incubated with IAV for 30 min, 37^°^C followed by addition of MUNANA substrate (provided with the kit). Samples were further incubated for another 1hr at 37^°^C. The reaction was then stopped and read using a POLARstar OPTIMA fluorescent plate reader (BMG Labtech, Durham NC). The NA activity was expressed as % of control. Raw fluorescence data are provided under S1 Data.

### Measurement of viral RNA

RNA for the viral M protein was measured using real time PCR (qPCR) as previously described [8]. MDCK cells were infected with IAV virus strains incubated for 30 min at 37^°^C with or without various doses of GI-20 or LL-37. RNA extraction was done at 45 min and 24 hrs post infection using Magmax viral RNA isolation kit (Applied Biosytems, Carlsbad, California) as per manufacturer’s instructions. Both lysed cells and cell supernatant were used for extraction. Viral RNA was also extracted from different concentrations of virus with known FFC/ml which was used as standard series. RNA was reverse transcribed using TaqMan reverse transcription reagents (Applied Biosytems, Carlsbad, California). The reaction mix and the cycle conditions were as per manufacturer’s instructions. For real time PCR, primers specific for IAV M protein (Forward AGA CCA ATC CTG TCA CCT CTGA and Reverse: CTG CAG TCC TCG CTC ACT) were used. The primers and TaqMan-labelled probes with non-fluorescent minor groove binder (MGB) moieties were designed manually using the Primer Express software version 3.0 (Applied Biosystems, Carlsbad, California) and were also synthesized by Applied Biosystems.

### Statistics

Statistical comparisons were made using Student's unpaired, two-tailed *t* test or ANOVA with post hoc test (Tukey’s). ANOVA was used for multiple comparisons to a single control. P values less than or equal to 0.05 were considered significant.

## Results

### Antiviral activity of LL-37 and derived fragments against seasonal H3N2 IAV and mouse-adapted PR-8 H1N1 IAV

**Fig 2:**
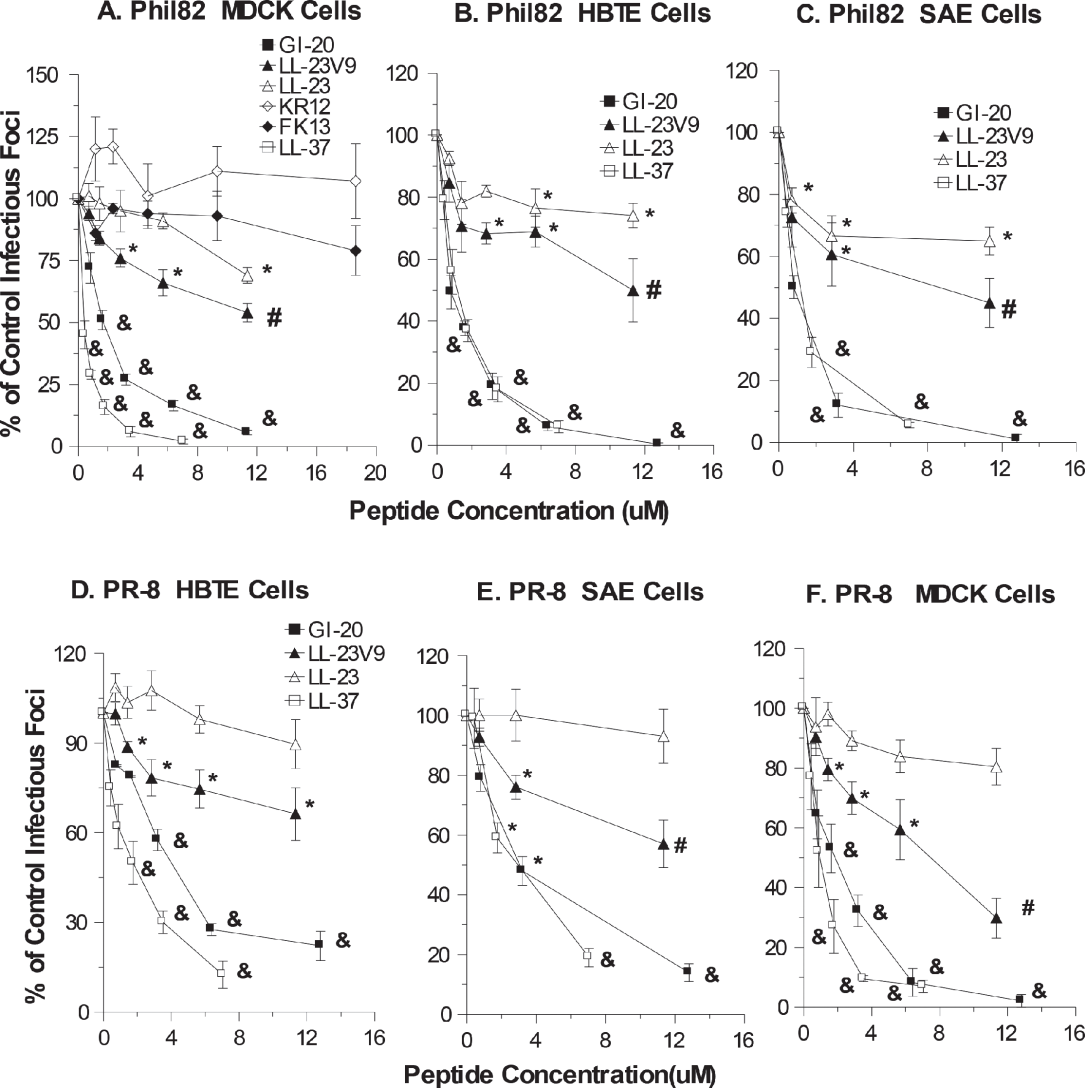
Effects of LL-37 and derived peptides on replication of Phil82 and PR-8 viral strains. For panels A-C, aliquots of the Phil82 H3N2 IAV strain were pre-incubated with control buffer (PBS) or the indicated concentrations of LL-37, FK-13, KR-12, LL-23, LL-23V9, or GI-20 peptides and then these samples were used to infect epithelial cell monolayers and tested for infectious foci 24 hrs later using anti-nucleoprotein antibodies and fluorescence detection (see Methods). Panel A shows activity of all of the peptides in MDCK cells. Panels B and C show activity of LL-37, LL-23, LL-23V9, and GI-20 in human bronchial/tracheal (HBTE) and small airway (SAE) epithelial cells, respectively. Panels D-F show results of similar experiments done using the PR-8 virus. LL-23 did not cause inhibition of PR-8 in these experiments. LL-23V9 caused modest inhibition of the virus. Inhibition by LL-23V9 again was significantly greater than LL-23 and LL-37 and GI-20 caused significantly greater inhibition than either LL-23 or LL-23V9. The average number of infected cells per well were 141, 100 and 78 for MDCK, HTBE, and SAE cells, respectively. Results are mean±SEM of 4 or more experiments and expressed as mean±SEM % of control infectious foci. Please refer to S1 Data for raw data for this and other figures. * indicates p<0.05 vs control buffer alone (unpaired t test). & indicates p<0.05 for LL-37 or GI-20 compared with other peptides and control (ANOVA analysis). # indicates where LL-23V9 caused significantly greater inhibition than LL-23 (ANOVA analysis).

Fig 1 depicts the different LL-37 derived peptides used in this study. Although LL-37 had clear dose-related antiviral activity against the seasonal Phil82 H3N2 strain of IAV as reported, the FK13 and KR12 fragments of LL-37 were without neutralizing activity (Fig 2A). The LL-23 fragment had slight antiviral activity against Phil82. The LL-23V9 peptide had significantly increased activity compared to LL-23; however, GI-20, the central fragment of LL-37, had increased activity against Phil82 as compared to either LL-23 or LL-23V9. The activity of GI-20 approached or equaled that of full length LL-37 in these assays. We performed LDH assays to determine if the peptides had any effect on viability of the MDCK cells under the same conditions as the neutralization assay (Table 1). No significant increase in cytotoxicity was observed.

**Table 1:** Effects of LL-37 derived peptides on cell viability as assessed by LDH assay.

	% Viability of MDCK or HBTE Cells^a^					
Cell Type	Peptide Concentration (μM)	LL-37	LL-23	LL-23V9	GI-20	sLL-37
**MDCK+IAV**	**0**	104±6	105±4	105±4	105±4	104±6
**MDCK+IAV**	**12.8**	99±12	101±11	108±7	107±3	106±1
**MDCK no IAV**	**12.8**	92±11	105±4	112±6	103±7	109±4
**HBTE + IAV**	**0**	97±2	97±2	97±2	97±2	97±2
**HBTE+IAV**	**3.2**	105±4				99±4
**HBTE+IAV**	**6.4**	91±4				99±2
**HBTE+IAV**	**12.8**	55±6*	98±3	99	104±3	97±4
**HBTE no IAV**	**3.2**	101±3				96±2
**HBTE no IAV**	**6.4**	95±2				96±1
**HBTE no IAV**	**12.8**	57±6*	98±1	103	103±3	103±2

^**a**^ MDCK or HBTE were treated 12μM of the indicated peptides along with virus (Phil82 strain) or without virus. The experiments were carried out in the identical manner as the infectious focus or qPCR assays. The percent of viable cells was measured using an LDH assay as described in methods. Where indicated by a * samples had significantly reduced viability compared to control. Results are mean±SEM of 3 or 4 experiments, except in the case of LL-23V9 and HBTE cells where n = 1.

To determine if the antiviral activities observed also occur in primary respiratory epithelial cells we compared LL-37, LL-23, LL-23V9 and GI-20 using HBTE and SAE cells. Of note, the number of infected cells in the HBTE or SAE cultures were consistently less in these and subsequent experiments despite use of the same starting virus concentrations. In any case, similar relative antiviral activities for the three peptides were found in these cells (Fig 2 panels B and C).

We also tested LL-37, LL-23, LL-23V9 and GI-20 for ability to inhibit hemagglutination activity of Phil82 IAV. No inhibition was observed at concentrations up to 12μM for any of these peptides in three experiments. This is consistent with our prior findings with other anti-microbial peptides (e.g. human neutrophil defensins) [24, 31].

**Fig 3:**
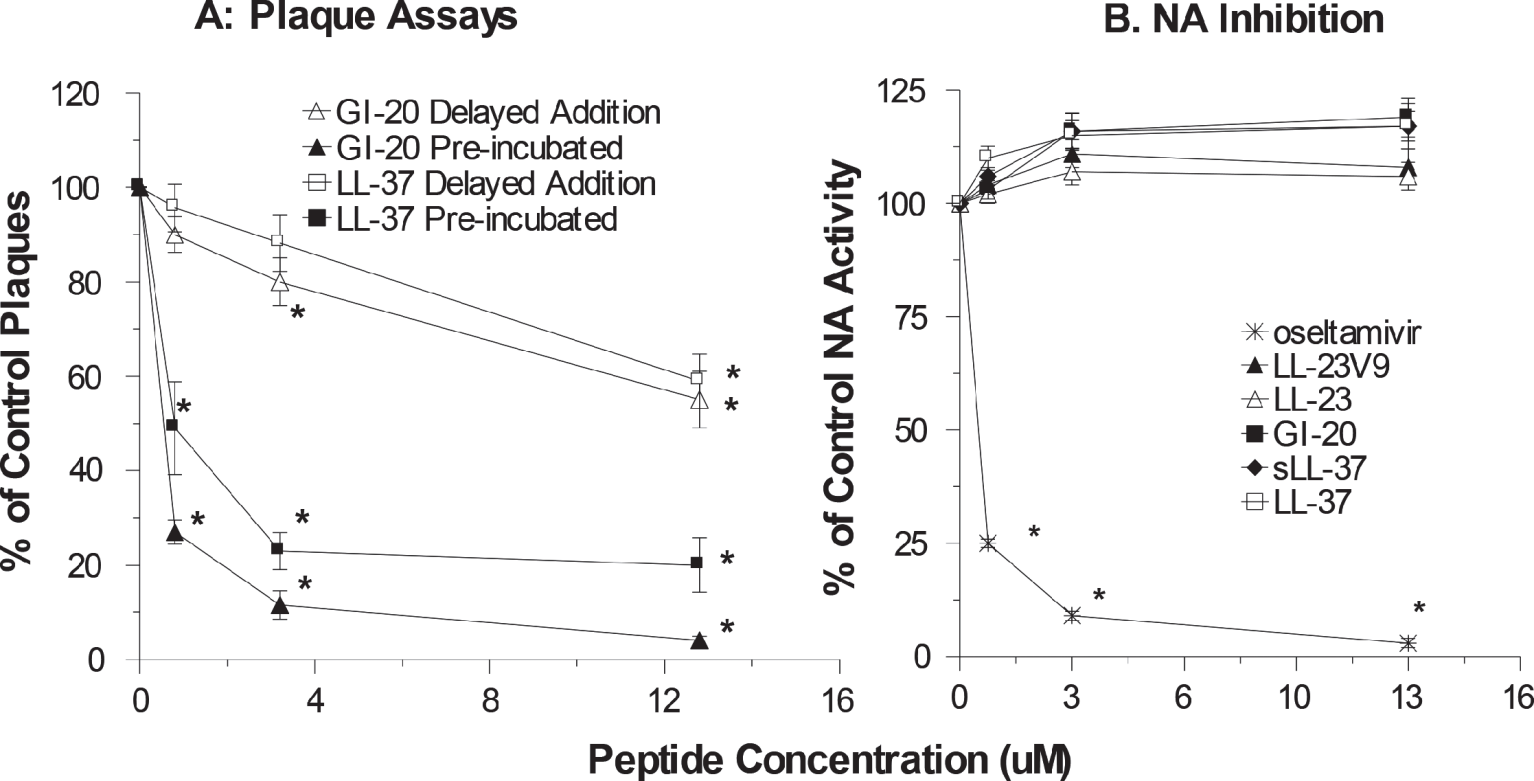
Effects of LL-37 on plaque assays and neuraminidase inhibition with Phil82 IAV. Panel A compares the ability of LL-37 and GI-20 to inhibit plaque formation by Phil82 IAV. The virus was either pre-incubated with the peptides prior to the assay, or the peptides were added to the monolayers after allowing 45 min for virus to infect the cells (“Delayed Addition”). Panel B shows neuraminidase inhibition assay results using LL-37, sLL-37, or LL-37-related peptides. Phil82 virus was pre-incubated with the peptides and then NA activity was assayed as described in Materials and Methods. Oseltamivir was used as a positive control and it caused marked reduction of NA activity. Results are mean±SEM of 4 or more experiments. * indicates p<0.05 vs control buffer alone (unpaired t test).

As shown in Fig 2 panels D-F, LL-23, LL23V9, and GI-20 had similar relative activities against PR-8 as against Phil82. Once again LL-23 had only slight neutralizing activity in MDCK, HBTE or SAE cells, LL-23V9 had somewhat greater activity, and the GI-20 had the greatest activity among the fragments of LL-37. We also performed plaque assays to confirm that GI-20 and LL-37 had comparable antiviral activity. The plaque assay differs from the infectious focus assay mainly in allowing for repeated rounds of viral replication. As shown in Fig 3A, LL-37 and GI-20 had very similar inhibitory activity for the Phil82 IAV strain in this assay. Using this assay we also tested the effect of adding the peptides after initial infection of the cells with virus. The inhibitory activity was markedly reduced using this method. In addition, we tested the ability of the peptides to inhibit neuraminidase (NA) activity of Phil82using the MUNANA fluorescence assay. As shown in Fig 3B, neither LL-37 nor the related peptides inhibited NA activity.

### Effects of LL-37 on replication of seasonal or pandemic H1N1 IAV in epithelial cells

**Fig 4:**
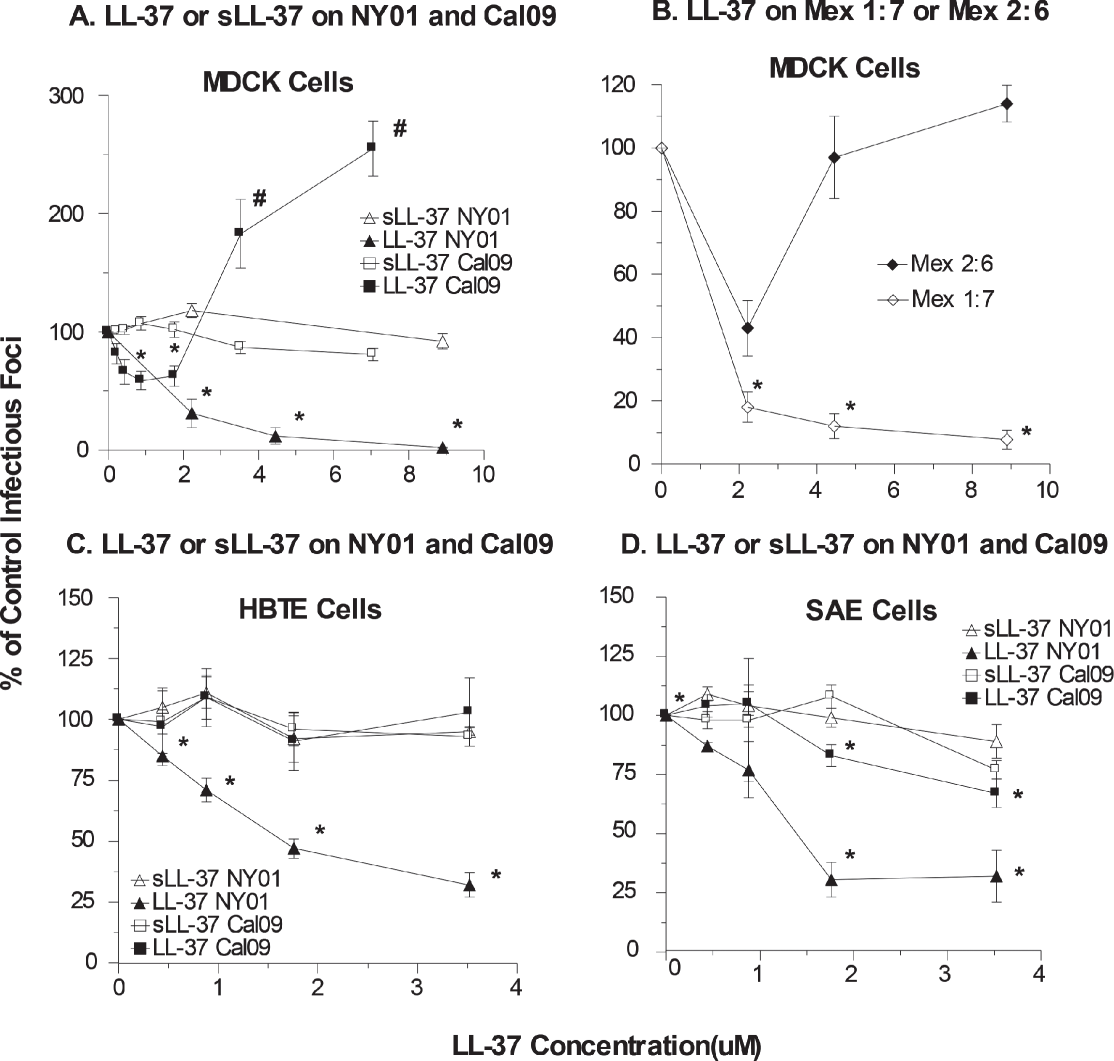
Effects of LL-37 or sLL-37 on replication of seasonal or pandemic H1N1. The effects of LL-37 or sLL-37 on the indicated H1N1 strains were tested using the infectious focus assay as in Fig 2. Panel A shows results with the Cal09 pandemic H1N1 strain Cal09 and the seasonal H1N1 strain NY01. Panel B compares effects if LL-37 on two additional recombinant H1N1 strains that had the HA only (Mex 1:7) or HA and NA (Mex 2:6) of the Mex09 pandemic strain combined with the other gene segments from NY01. Panels C and D show results of similar experiments using HBTE and SAE cells, respectively. Results are mean±SEM of 4 or more experiments. * indicates a significant decrease in viral foci (p<0.05) compared to control. # indicates a significant increase in viral foci (p<0.05) compared to control.

We used the Cal09 H1N1 strain from the 2009 pandemic to test the activity of LL-37. We expected to find that LL-37 would inhibit this strain since it had comparable activity against all the viral strains tested thus far. Surprisingly only slight inhibition at intermediate doses of LL-37 and actual enhancement of the replication of this strain at higher doses in MDCK cells (Fig 4A). A scrambled LL-37 (sLL-37) control had no effect on replication of Cal09. Since this strain was derived by reverse genetics and propagated in MDCK cells rather than eggs, we compared the effects of LL-37 on a seasonal IAV strain (NY01) developed and propagated in the same manner as Cal09. LL-37 caused clear dose related inhibition of the NY01 strain (Fig 4A). Again sLL-37 had no activity vs NY01.

To evaluate this effect further we tested the activity of LL-37 against two recombinant strains, one of which contained the hemagglutinin (HA) and neuraminidase (NA) proteins of the Mex09 H1N1 strain (Mex 2:6) and one with just the HA of Mex09 (Mex 1:7). The Mex09 HA and NA proteins are nearly identical to those of Cal09. The remaining proteins of the recombinant strains were contributed by NY01. As shown in Fig 4B the Mex 2:6 strain was inhibited by LL-37 at the lower concentration of 2.2μM but higher concentrations of LL-37 were not inhibitory. Of interest, the Mex 1:7 (having only the HA of Mex09) was inhibited at all concentrations tested.

In HBTE cells LL-37 did not inhibit infectivity of Cal09, while again inhibiting NY01 in parallel. In these cells LL-37 did not paradoxically increase infectivity of Cal09 (Fig 4C). LL-37 caused slight (but statistically significant, p<0.05) inhibition of Cal09 (Fig 4D) in SAE cells. However, NY01 was significantly more inhibited than Cal09 in SAE cells.

**Fig 5:**
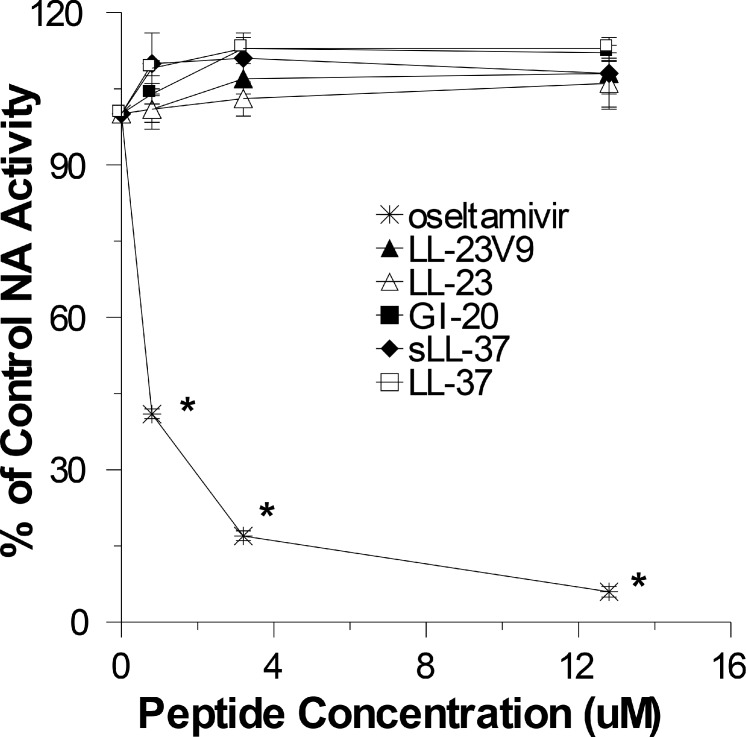
Effects of LL-37 and related peptides on neuraminidase activity of Cal09. The figures shows effects of LL-37, sLL-37 or related peptides on NA activity of Cal09. NA activity was measured as in Fig 3. Results are mean±SEM of 4 or more experiments.* indicates a significant decrease in viral foci or NA activity (p<0.05) compared to control.

Since the experiments with the Mex09 derived strains suggested a role for the pandemic NA in resistance of pandemic H1N1 to LL-37, we also tested the ability of LL-37, sLL-37 or related peptides to inhibit NA activity of Cal09 as shown in Fig 5. As with the seasonal Phil82 strain, the peptides did not inhibit NA activity of the pandemic strains.

### Effects of LL-37 on viral uptake by MDCK cells or viral RNA synthesis in these cells as assessed by qPCR

**Fig 6:**
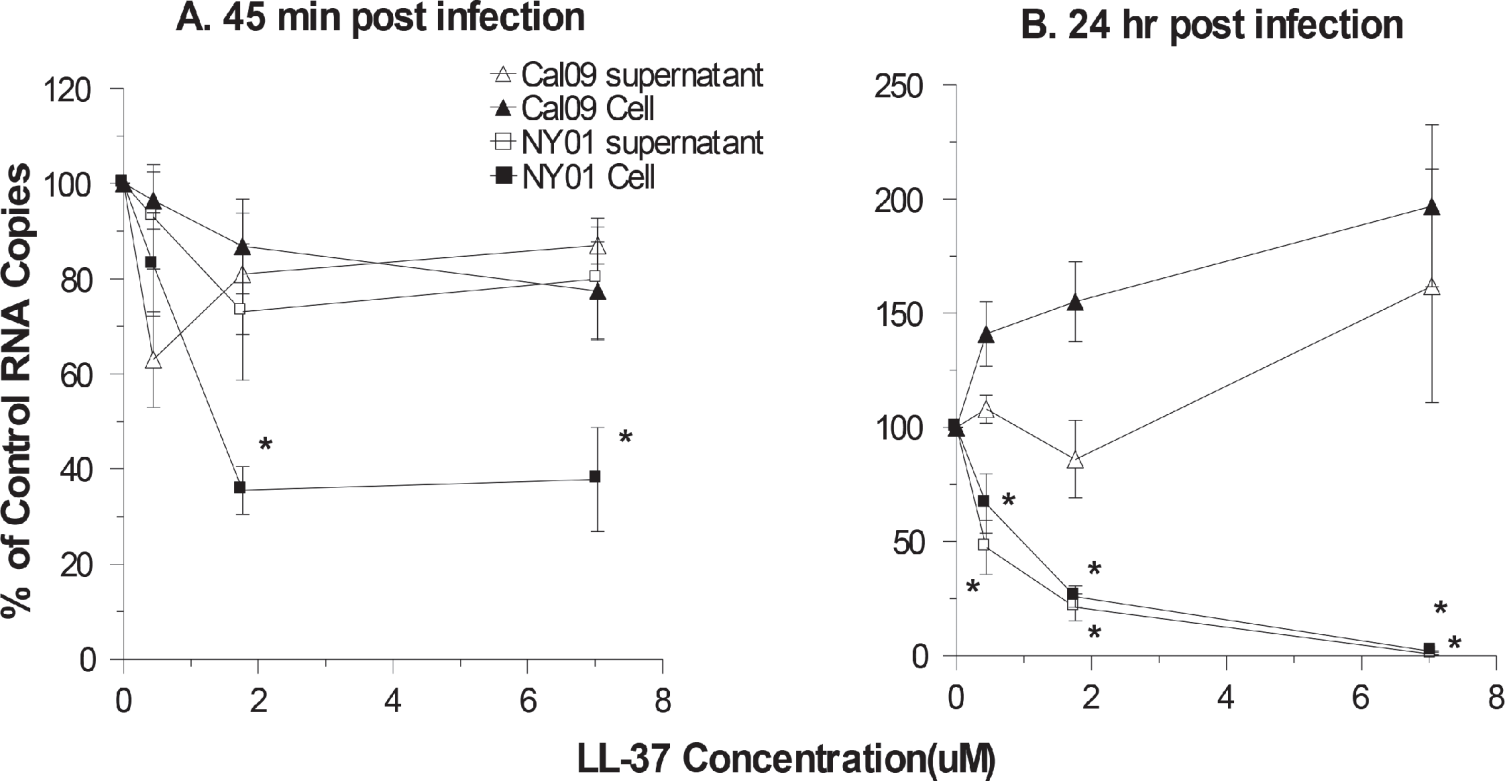
Effects of LL-37 on viral replication as assessed by quantitative RT-PCR. Results shown are percent of control viral RNA copy numbers for samples treated with LL-37 or sLL-37. Panel A shows amounts of cell-associated or supernatant virus after 45 min of incubation with MDCK cells. There were no significant differences in viral RNA quantities between control and LL-37 treated samples in panel A except for cell associated NY01 (* indicates p<0.05 vs control). Panel B shows results in which RNA was isolated from cells and supernatants after 24 hours of infection. LL-37 at concentrations ≥0.4μM significantly decreased viral RNA in supernatants and cells for NY01 strain but not for the Cal09 strain in panel B. All results are mean±SEM of 4 or more separate experiments.

We next used qPCR to confirm the findings of the infectious focus assay through an independent assay. This was done by pre-incubating the virus with different concentrations of LL-37, and then incubating the viral samples with the cells for 45 min at 37°C. We first tested the effects of LL-37 on viral uptake by the MDCK cells. This was done by harvesting cells and supernatants were separately after the 45 min incubation and assaying both for quantities of RNA of the viral M protein. As shown in Fig 6A, there was no significant difference in viral uptake of Cal09 at the different doses of LL-37 (see cell lysate results). There was an apparent increase in Cal09 RNA in the supernatant of the LL-37 treated cells but this was highly variable and not statistically significant. There was a significant reduction of uptake of the NY01 into cells in these experiments. Cells and supernatant were next assayed for presence of viral RNA at 24 hrs after infection. In this case there was a significant reduction in RNA of NY01 in both cells and supernatant (Fig 6B). There was a trend to increase of Cal09 in the cells in presence of LL-37 but this was again highly variable and not statistically significant. These results confirm the reduced activity of LL-37 to inhibit the pandemic strain in these cells.

### Effects of CRAMP and HNP-1 on replication of pandemic H1N1

**Fig 7:**
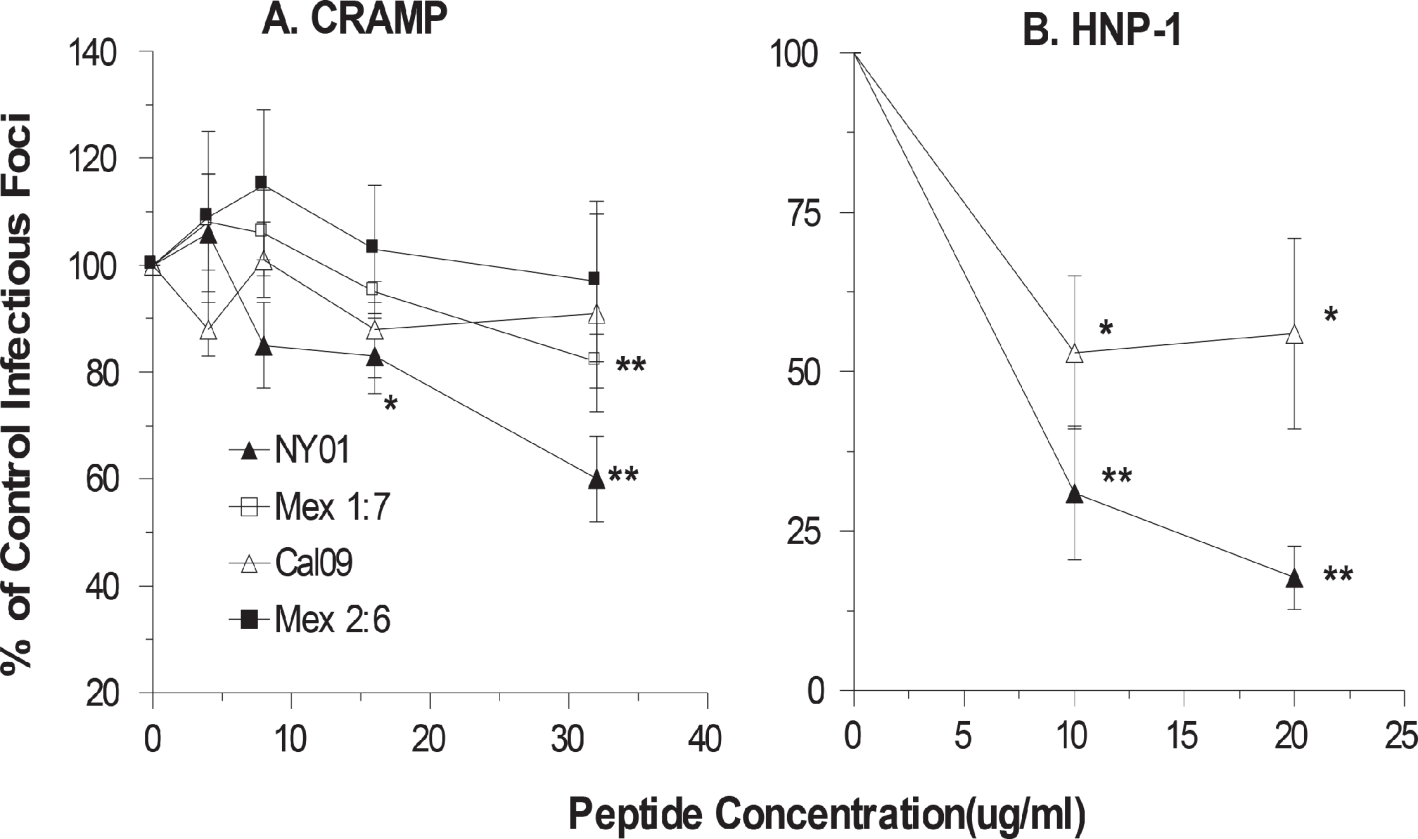
Effects of CRAMP and HNP-1 on replication of seasonal or pandemic H1N1 strains. The effect of pre-incubating Cal09, NY01, Mex 1:7, or Mex 2:6 H1N1 strains on infectivity was assayed as in Fig 5 using the infectious focus assay. Results are mean±SEM of 4 or more experiments. * indicates p<0.05 vs control buffer. ** indicates p<0.01 vs control buffer.

To determine if the lack of antiviral activity of LL-37 against pandemic H1N1 applied to other cationic antimicrobial peptides we tested the murine cathelicidin, CRAMP, and the human defensin HNP-1, against Cal09, Mex2:6, Mex1:7 and NY01. As shown in Fig 7A, CRAMP behaved like LL-37 in that it did not significantly inhibit CAL09 or Mex2:6 and inhibited (albeit modestly) Mex1:7 and NY01. Note the inhibitory activity of CRAMP against NY01 was less pronounced than that of LL-37 which is similar to our prior results using other seasonal viruses [8]. HNP-1 did inhibit Cal09 (Fig 7B) although the activity was attenuated compared to its activity against NY01.

### Effects of LL-37-related peptides on replication of pandemic H1N1

**Fig 8:**
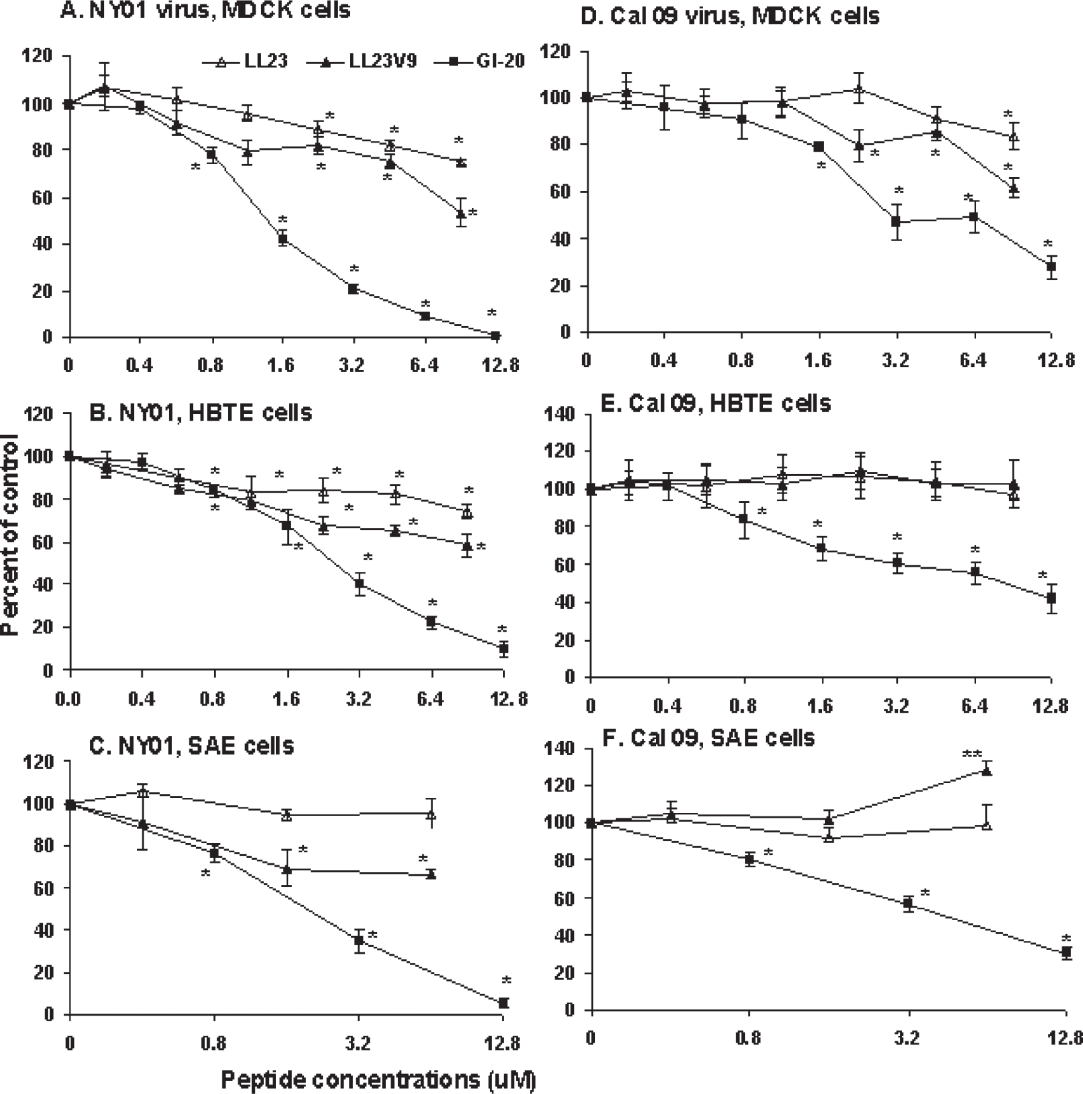
Effects of LL-23, LL-23V9 or GI-20 on replication of Cal09 or NY01 in various cell types. All of the peptides caused significant inhibition of NY01 in MDCK cells and HBTE cells. For SAE cells LL-23V9 and GI-20 caused significant inhibition for NY01 but LL-23 did not. GI-20 caused significant inhibition of Cal09 in all three cell types. LL-23 and LL-23V9 caused modest but statistically significant inhibition of Cal09 in MDCK cells but not in HBTE or SAE cells. LL-23 (11μM) caused significant increase in replication of Cal09 in SAE cells. Results are mean±SEM of 4 or more experiments. * indicates a significant decrease in viral foci (p<0.05) compared to control. ** indicates a significant increase in viral foci (p<0.01) compared to control.

Using the infectious focus assay again (as in Fig 2) we found very similar results using NY01 as we obtained with Phil82 and PR-8: LL-23 had limited or no (in case of SAE cells) antiviral activity, activity of LL-23V9 was somewhat greater, and the GI-20 fragment had the strongest activity (Fig 8A–8C). Results obtained with Cal09 were somewhat cell type dependent. All three peptides had some activity against Cal09 in MDCK cells with the central fragment having the most notable activity (Fig 8D). For HBTE and SAE cells GI-20 retained fairly strong activity against Cal09 but LL-23 and LL-23V were without activity (Fig 8E and 8F). LL-23 actually increased viral replication of Cal09 in SAE cells to a limited extent.

## Discussion

### Activity of LL-37 or derived peptides against seasonal strains of IAV and the PR-8 mouse adapted strain

Table 2 shows the comparative neutralizing activity of various antimicrobial peptides for different IAV strains (incorporating results from this and prior papers). The results are given in both μg/ml and μM amounts for comparison. Several fragments of LL-37 that have been reported to have some activity against bacteria had limited or no activity against IAV, including FK-13, KR-12 and LL-23. The lack of activity of FK-13 is notable since this peptide has inhibitory activity against HIV-1 [15], and both FK-13 and KR-12 have activity against bacteria [14]. Of interest the LL-23V9 had consistently increased activity compared with LL-23 vs. several IAV strains and in different cell types. This is consistent with previous finding that this modified peptide has increased activity against bacteria [13]. It also suggests that having a continuous hydrophobic surface on the peptide is important for antiviral as well as antibacterial activity (Fig 1). Nonetheless the LL-23V9 peptide did not have as much activity as full length LL-37. In contrast, GI-20, the central fragment corresponding to the central helix of LL-37 (Fig 1), had strong antiviral activity in all of our experiments. The only difference of GI-20 from the central 20 amino acids of LL-37 is inversion of the IG sequence of amino acids 13 and 14 (see Fig 1). Overall our results indicate that the central helix of LL-37 is required for optimal anti-IAV activity since shortened peptides FK-13 and KR-12 are poorly active (Fig 2)

**Table 2:** Comparison of approximate 50% viral neutralizing concentrations of antimicrobial peptides in MDCK cells.

	LL-37	LL-23	LL-23V9	GI-20	HNP-1
**Phil82 H3N2**	~2μg/ml (~0.44 μM)	>32μg/ml (>11.3 μM)	~32μg/ml (>11.3 μM)	~4μg/ml (~1.6 μM)	~2.5μg/ml (~0.44 μM)^a^
**PR-8 H1N1**	~4μg/ml (~0.89 μM)	>32μg/ml (>11.3 μM)	~16μg/ml (~5.7 μM)	~4μg/ml (~1.6 μM)	~1.9μg/ml (~0.29 μM) ^a^
**Cal09 H1N1**	>32 μg/ml (>11.3 μM)	>32μg/ml (>11.3 μM)	>32μg/ml (>11.3 μM)	~8μg/ml (~ 3.2 μM)	
**NY01 H1N1**	~5μg/ml (~1.1 μM)	>32μg/ml (>11.3 μM)	~32μg/ml (~11.3 μM)	~4μg/ml (~1.6 μM)	

^a^ Results taken from [31]

### Failure of LL-37 to inhibit pandemic H1N1

Another important finding of this paper is that LL-37 has minimal or no inhibitory activity for the Cal09 pandemic strain in MDCK, HBTE or SAE cells, while in each case there was clearly greater inhibition of the seasonal NY01 strain prepared in a similar manner. We used qPCR to show that LL-37 did not alter uptake of Cal09 by MDCK cells and to confirm the lack of inhibitory activity of LL-37 for this strain. A viral strain having only the HA and NA of pandemic H1N1 of 2009 (Mex 2:6) in combination with the six other viral gene segments of seasonal H1N1 (NY01) was partially inhibited at a lower concentration of LL-37 in MDCK cells, but this inhibition was again lost at higher concentrations of LL-37. However, a strain containing only the pandemic HA (Mex 1:7) was inhibited by LL-37. These results suggest the effects of LL-37 are not determined by interaction with the viral HA. This is consistent with the finding that LL-37 does not inhibit viral hemagglutination activity [8]. The results also suggest that the pandemic NA may be important in mediating effects of LL-37. Of note, however, we also show that LL-37 does not inhibit NA activity of seasonal or pandemic IAV strains. Further research with additional recombinant viral strains and additional assays will be needed to elucidate the mechanism of these findings. It is likely that other genes of the pandemic virus are involved in resistance to LL-37 since the results obtained with Mex 2:6 in MDCK cells differed somewhat from those obtained with Cal09 (e.g., Mex 2:6 was inhibited more at the lower concentrations and infectivity was not increased as much at higher concentrations). CRAMP also lacked the ability to inhibit Cal09 or the Mex2:6 strain and inhibited Mex 1:7 and NY01. HNP-1 had reduced inhibitory activity for Cal09 as compared to its activity against NY01. These results suggest that the resistance of Cal09 may apply to a variety of cationic antimicrobial peptides.

Our finding that cationic anti-microbial peptides have reduced activity against pandemic H1N1 fits in with a larger theme in which pandemic IAV is resistant to other innate inhibitors including collectins and pentraxins [16, 17]. In the case of the collectins, surfactant protein D and mannose binding lectin, the resistance applies to all recent pandemic strains and relates to reduced glycosylation on the HA of these strains. Surfactant protein A also has limited activity against Cal09 as compared to its activity against other viral strains [32]. SP-A has a distinct mechanism of inhibiting IAV compared to the other collectins [33]. In this case SP-A does not use its lectin activity to bind to HA-associated glycans but rather it provides a sialylated glycan ligand to which the HA can bind. Pentraxin has a similar mechanism as SP-A [16, 34]. This mechanism has been termed γ-inhibition. H-ficolin also functions as a γ-inhibitor vis a vis IAV but it is able to inhibit Cal09 H1N1 [32]. The antiviral mechanism of LL-37 is not fully defined but it does not involve HA inhibition, viral aggregation or inhibition of IAV uptake by epithelial cells (at least in the case of Phil82) [8], which distinguishes it from these other inhibitors. Hence, Cal09 shows in vitro resistance to a range of innate inhibitors that have distinct mechanisms of action. The 1918 H1N1 pandemic strain and H2N2 pandemic strain were also not inhibited by SP-D [17]. These findings suggest that one of the reasons for the increased pathogenicity of pandemic H1N1 is its ability to bypass some initial soluble host defense barriers.

### The central fragment of LL-37 has inhibitory activity for Cal09

An additional notable finding of this paper is that the central fragment GI-20 had greater activity against Cal09 than LL-37 in all cell types tested (Fig 8). This result provides basis for further development and testing in vivo of this peptide. In addition, prior studies have shown that GI-20 has strong anti-HIV activity with the best therapeutic index among a library of LL-37-derived peptides, including LL-23, FK-13, and KR-12 [15].

### Conclusions

Taken together, the central fragment of human LL-37 is essential for optimal antiviral activity and constitutes a useful template for peptide engineering to boost human host defense. Further engineering work is ongoing in Dr. Wang's laboratory based on this patented template (Wang, G. Anti-HIV Peptides and Methods of Use Thereof, US 20120237501 A1). It is of particular interest that pandemic H1N1 was found to be resistant to antiviral activities of LL-37, CRAMP and (to an extent) HNP-1 and that this resistance is overcome by GI-20. In future studies we will also explore the mechanism of antiviral activity of GI-20 and the immune modulatory effects of the LL-37 derived peptides with respect to IAV. This will be important since LL-37 has been found to have important immuno-modulatory effects during IAV infection in vivo [4].

## Supporting Information

S1 Data Supporting Information for the article. (XLS)
